# Membrane Phospholipid Augments Cytochrome P4501a Enzymatic Activity by Modulating Structural Conformation during Detoxification of Xenobiotics

**DOI:** 10.1371/journal.pone.0057919

**Published:** 2013-02-28

**Authors:** Manik C. Ghosh, Arun K. Ray

**Affiliations:** 1 Department of Physiology,University of Tennessee Health Science Center, Memphis, Tennessee, United States of America; 2 Division of Molecular Medicine (formerly, Dept. of Animal Physiology), Bose Institute, Calcutta, West Bengal, India; Oak Ridge National Laboratory, United States of America

## Abstract

Cytochrome P450 is a superfamily of membrane-bound hemoprotein that gets involved with the degradation of xenobiotics and internal metabolites. Accumulated body of evidence indicates that phospholipids play a crucial role in determining the enzymatic activity of cytochrome P450 in the microenvironment by modulating its structure during detoxification; however, the structure-function relationship of cytochrome P4501A, a family of enzymes responsible for degrading lipophilic aromatic hydrocarbons, is still not well defined. Inducibility of cytochrome P4501A in cultured catfish hepatocytes in response to carbofuran, a widely used pesticide around the world, was studied earlier in our laboratory. In this present investigation, we observed that treating catfish with carbofuran augmented total phospholipid in the liver. We examined the role of phospholipid on the of cytochrome P4501A-marker enzyme which is known as ethoxyresorufin-O-deethylase (EROD) in the context of structure and function. We purified the carbofuran-induced cytochrome P4501A protein from catfish liver. Subsequently, we examined the enzymatic activity of purified P4501A protein in the presence of phospholipid, and studied how the structure of purified protein was influenced in the phospholipid environment. Membrane phospholipid appeared to accelerate the enzymatic activity of EROD by changing its structural conformation and thus controlling the detoxification of xenobiotics. Our study revealed the missing link of how the cytochrome P450 restores its enzymatic activity by changing its structural conformation in the phospholipid microenvironment.

## Introduction

Cytochrome P450 belongs to a broad spectrum enzymatic systems responsible for detoxifying endogenous substrates, such as, steroids and hormones, as well as exogenous compounds, including drugs and lipophilic hydrocarbons [1]. Among all P450s, cytochrome P4501A (CYP1A) is a family of protein that is reportedly involved in degrading the lipophilic substrate known as polycyclic aromatic hydrocarbons [2]. Several reports have indicated that enzymatic activity of cytochrome P450 protein can be enhanced in the phospholipid environment as evidenced by augmented enzymatic activity in presence of dilauylphosphatidyl choline (DLPC*)* [3], [4], [5], [6]. The ability of phospholipid in modulating an enzymatic structural conformation was demonstrated by many workers [4], [6], [7], [8], [9], [10], [11], [12]. How structural conformation was intricately involved in regulating the enzymatic activity had elegantly demonstrated by several other authors [13], [14], [15], [16], [17]. Circular dichroism (CD) is a powerful spectroscopic method used to unravel the behavior and folding pattern of P450s and their interactions with the lipid-lipid bilayer [18]. The conformational studies on P450s from fish with CD has not yet been reported, although it is immensely important as the simplified structure-function relationship in this lower vertebrate may unveil the biochemical and biophysical behavior of this protein during toxic stress in physiological microenvironment.

We sought to investigate the biochemical activity of purified cytochrome P4501A protein containing ethoxyresorufin-O-deethylase (EROD) activity because compelling evidence indicated that phospholipid could encompass a significant impact on restoring the enzymatic activity of cytochrome P450 protein [10], [19], [20], [21]. We reported earlier that carbofuran could be metabolized by microsomal protein, such as, the cytochrome P4501A enzymatic system in catfish liver [22]. Carbofuran is reported to induce the total phospholipid in the liver in mammals and fish [23], [24]. We observed that treating catfish with carbofuran triggered a significant upregulation of total phospholipid in the hepatocyte membrane but we could not understand how this phospholipid was involved in alteration of cytochrome P450 enzymatic activity. We purified carbofuran induced-cytochrome P4501A protein from catfish liver and examined the role of this phospholipid on the structure and function of purified protein, including its catalytic activity. Our data demonstrated that purified protein which was devoid of enzymatic activity could restore its partial enzymatic activity in the presence of phospholipid. The structural study of this protein with circular dichroism (CD) confirmed that phospholipid could orchestrate the folding of the protein and thereby providing an impetus to regain its enzymatic activity. The biophysical and biochemical characterization revealed a detailed understanding of cytochrome P4501A enzymatic activity for the *in situ* microenvironment during detoxification of xenobiotics.

## Materials and Methods

Diethyl amino ethyl cellulose (DEAE, commercial name, DE-52), an anion exchanger was purchased from Whatman (England), hydroxyapatite, CHAPS, Na-cholate, phenylmethylsulphonyl fluoride (PMSF), leupeptin, EDTA, glycerol, and dithiothreitol (DTT) were purchased from Sigma-Aldrich Chemicals (St. Louis, MO). Carbofuran was obtained as a generous gift from Rallis India, Karnataka. Reagents for SDS gel electrophoresis, Western blot, ELISA, and dot-blot were purchased from GIBCO-BRL (Carlsbad, CA).

### Injection of Carbofuran and Collection of Liver from Fish

Maintenance of catfish used in this study was performed in an institute aquarium. Treatment, sacrifice, and collection of livers from fish were undertaken with the approval of the Bose Institute Animal Ethics Committee (Protocol # 95/99/CPCSEA). Isolating and culturing hepatocytes from catfish livers were performed following the methods routinely used in our laboratory [22], [25].

Catfish (*Heteropneustes fossilis*) were purchased from the local market (average body weight 100 g) and acclimatized to laboratory conditions for 7 days with food given ad libitum into an aquarium with circulating water. Fish were injected with three repeated intraperitoneal injections of carbofuran (CF), 0.1 µg/gm body weight, at 0 h, 24 hrs, and 48 hrs and sacrificed at 96 hrs. Fish were sacrificed by a single blow to the head, and livers were collected in ice-cold, 20 mM potassium phosphate buffer (pH 7.4) with 0.25 M sucrose, 1 mM PMSF, 1 mM DTT, 1 mM leupeptin, 1 mM EDTA, 10% glycerol (Buffer-I), and washed several times with the same buffer to minimize blood mixing. After removing cell debris, microsome were prepared by ultracentrifugation (100,000×g) and stored at −80°C for future use.

### Extracting and Measuring Total Lipid and Phospholipid from Membrane Fraction of Liver

Total lipid was extracted from microsome according to the method of Bligh and Dyer [26]. In brief, the microsome was dissolved in 6 ml of chloroform and methanol solution (1∶2) and agitated to obtain homogenous solution to which 1.6 ml of water was added. Then solution was transferred to glass screw cap tube and placed at 4°C overnight. There was a clear phase separation. The organic part was collected and residue of solvent was evaporated with the help of nitrogen steam. The amount of phospholipid was estimated from the total lipid according to the method of Rouser et al. [27]. The principle of estimating the phospholipid is based upon the calculation of presence of inorganic phosphate in total lipid.

The standard was prepared from KH_2_PO_4._ In brief, lipid samples are transferred into clean glass tubes and 0.65 ml perchloric acid was added. The tubes were placed on the heated block for about 30 min until the yellow color has disappeared. When cool, 3.3 ml water, 0.5 ml of molybdate solution, and 0.5 ml of ascorbic acid were added sequentially. Solution was agitated on a vortex after each addition. Tubes were placed in a boiling water bath for 5 min. The absorbance of cool samples was read at 800 nm with the help of a spectrophotometer. Standards solution of KH2PO4 (1, 2, 4, 8, 10 and 20 µg inorganic phosphate/tube) were treated as similar way performed for the unknown samples. The amount of total phospholipids was calculated directly from the amount of inorganic phosphate (Pi) present in standard.

### Solubilizing Microsomes

Microsomes were thawed and solubilized by drop-wise addition of 3-[(3-Cholamidopropyl) dimethylammonio]-1- propanesulfonate (CHAPS) and Na-cholate in the Buffer-I to a final concentration of 1.0% and 0.2%, respectively. The solution was stirred on ice for 1 hr and centrifuged at 100,000 g for 75 min at 4°C. The supernatant was collected and stored in −20°C.

### Treating Supernatant with Polyethylene Glycol

Enriching cytochrome P450 in total protein was done by using polyethelene glycol 6000 (PEG-6000). A 50% stock solution of PEG was prepared and stored at 4°C. The supernatant was treated with 10%, 20%, and 30% PEG followed by centrifugation at 100,000× g for 1 hr at 4°C. The supernatant was diluted in 20 mM potassium phosphate buffer (pH 7.4) with 10% glycerol, 1 mM DTT, 0.1 mM EDTA, 1 mM PMSF,1 mM leupeptin, 1% CHAPS, and 0.5% Na-cholate (Buffer-II).

### Adding Cell lysate to DE-52 Column

DE-52 is an anion exchanger resin equilibrated with Buffer II (bed volume 100 ml). The crude cell lysate was loaded to the column followed by a thorough washing with Buffer-II until no visible color was eluted. It helped excluding the hemoglobin and unbound protein from the column.

### Eluting Cytochrome P450 from DE-52

Cytochrome P450 was eluted from the column by applying a linear gradient (0–500 mM) of KCl in Buffer-II at the rate of 1 ml/min. The fractions (10 ml) were collected using an automated fraction collector. The two elution peaks of cytochrome P450 and heme containing P450 were observed at 300 mM of KCl, having absorption maxima at 280 and 416 nm, respectively. Four pure fractions containing cytochrome P450 were collected and labeled A, B, C, and D in the order of their elution. Cytochrome P450 fractions demonstrated maximum absorption at 416 nm compared to any other fractions eluted from the column. Hence, Fraction -D showed the highest absorption at 280 and 416 nm. All fractions were dialyzed overnight separately in 4 liter of 20 mM potassium phosphate buffer (pH 7.4) containing 10% glycerol at 4°C with at least three changes.

### Biochemical Assay

Protein concentration was assayed by the bicinchonnic acid (BCA) method [28]. The purity of protein was checked in sodium dodecyl sulfate polyacrylamide gel electrophoresis (SDS-PAGE) under reduced and non-reduced conditions according to the method of Laemmli [29].

### Measuring 7-EROD Activity in a Reconstituted System of Fractions A–D

Reconstituting EROD activity of the eluate from the DE-52 column was performed. The standard reaction mixture contained 5 µg of purified cytochrome P450 protein and 10 ug of NADPH-dependent cytochrome P450 reductase from mammalian source (Sigma Chemicals, St. Louis, MO) with 0.5 µM DLPC and 0.1 M potassium phosphate buffer (pH 7.4). The mixture was sonicated with six pulses of 10 seconds each followed by adding 2 µM substrate of 7-ethoxyresorufin. The assay was performed at 37°C. The reaction was initiated by adding 5 µl of 100 mM NADPH to a final volume of 1 ml. The activity of 7-EROD was expressed in pmol resorufin/min/mg of protein. The purity of Fraction-D was also examined SDS-PAGE.

### Purifying P450 by Hydroxyapatite Column

Fraction-D pooled from the DE-52 column was applied to the hydroxyapatite column (bed volume 40 ml) for further purification. The column was equilibrated with 10 volumes of Buffer II, pH 7.4. Elution of cytochrome P450 was performed by stepwise increase of the strength of potassium phosphate buffer. Fractions containing cytochrome P450 enzymatic activity were pooled and dialyzed as previously described.

### Capillary Electrophoresis

Capillary electrophoresis of purified protein obtained from the hydroxyapatite column was performed using an amine capillary column (57 cm) with the Beckman capillary electrophoresis system, model 5010 (Fullerton, CA). A sample containing 1 µg total protein was loaded onto the column.

### Coomassie and Silver Stain of the Protein Gel

H1 elute was run in SDS-PAGE to check the purity and molecular weight. The resolved gel was stained with coomassie blue. In addition, to get the finer detail of protein band, silver stain of the gel was performed. The gel was stained with filtered coomassie solution (10% acetic acid, 40% methanol, 50% water, and 0.1% coomassie R-250 powder) for 1 hr followed by destained with destaining solution (components are same as coomassie solution but without any R250 powder).

Silver stain of the H1 eluate was performed using a kit obtained from Bio-Rad (Hercules, CA).

### Production of Antibody

The handling, care, and use of rabbits for experiments were undertaken in compliance with the regulatory rules of the Bose Institute Animal Ethics Committee. Antibody against purified P450 was produced in a white rabbit (6 months old). The protein was mixed with Freund’s complete adjuvent and injected intramuscularly in rabbit. Blood was drawn from the rabbit by catheterization of a marginal ear vein. Serum was collected from blood and antibody titer was checked by ELISA.

### Enzyme-linked Immunosorbant Assay

The titer and specificity of the antibody from rabbit were tested by enzyme-linked immunosorbent assay (ELISA). The microsomal protein (1 µg/well) was added to a 96-well plate and incubated overnight at 4°C. Nonspecific binding was blocked by 5% skim milk followed by adding antiserum obtained from rabbit’s blood in consecutive ½ dilution with crude serum as initial strength. Horseradish peroxidase-conjugated anti-goat rabbit serum (1∶5000) was used as a secondary antibody (Gibco-BRL, Carlsbad, CA). Hydrogen peroxidase and diaminobenzidine (DAB) were used as substrates and color was recorded by ELISA reader (Bio-Rad, Hercules, CA) at 450 nm.

### Dot-blot Zhybridization

The specificity of antibody raised in rabbit was also tested by dot-blot. The microsomal protein adhered to the nitrocellulose membrane strip with different concentrations (20, 10, 5, 2, and 1 µg) keeping the BSA as negative control. Non-specificity was blocked by 5% skim milk, and rabbit antiserum was used as a primary antibody (1∶1000). Goat anti-rabbit horseradish peroxidase-conjugated serum was used as secondary antibody (1∶5000) followed by addition of substrate hydrogen peroxidase and DAB. Brown color was developed and the reaction immediately stopped by distilled water. The membrane was scanned with the help of a scanner (BioRad, Japan).

### Hepatocyte Culture

Hepatocytes were cultured from the liver of *Heteropneustes fossilis* following the method routinely used in our laboratory [22]. Hepatocytes were treated with different doses of carbofuran. Treated hepatocytes were harvested and microsomes were prepared from the sonicated hepatocytes by ultracentrifugation at 100,000×g for 1 hr. Microsome protein was frozen at −80°C for immunoblotting.

### Western Blot

Protein lysates that were collected from carbofuran-treated hepatocytes were used for immunoblot. Protein (30 µg) was run on 8% SDS-PAGE according to the method of Laemmli (1970). Upon resolving protein was transferred to nitrocellulose membrane with a transblot apparatus (Bio-Rad, Hercules, CA). Anti-rabbit serum against the purified protein was used at 1∶1000 dilution overnight at 4°C. Goat anti-rabbit IgG labeled with horseradish peroxidase (HRP) was used as secondary antibody for 1 h at room temperature. Membrane was developed by adding substrate and scanned as mentioned above.

### Study of the Catalytic Activity of Purified P450 Protein in the Reconstituted System

In the reconstituted system, catalytic activity of purified protein was studied in presence of different concentrations of DLPC, a synthetic lipid used for reconstituting membrane-bound enzyme. A specific amount of purified protein was added to reaction mixture with graded lipid environment (0.5, 1.0, and 2 uM) and with a mammalian preparation of NADPH-cytochrome P450 reductase (10 ug/ml) (Sigma Chemicals, St. Louis, MO). The reaction was started by adding 2 uM of 7-ethoxyresorufin in 1 ml of total reaction mixture, and catalytic activity was studied by measuring the production of resorufin. The activity of 7-EROD was expressed as pmol resorufin/min/mg of protein.

### Circular Dichroism (CD) of the Purified Protein Used to Study Secondary Structure

Secondary structures of the reconstituted purified protein in lipid environment were studied by CD. One microgram of protein was suspended in 20 mM phosphate buffer (pH 7.4), and CD spectra were recorded at near-UV (320 nm) to far-UV (200 nm) ranges. Spectra were recorded on a Jasco J700 spectropolarimeter (Japan Spectroscopic, Tokyo). The computation of the α-helicity from CD spectra was performed according to the method described earlier [30]. Mean residue amino acid molecular weight was considered as 113 as determined according to the method of [31].

## Results

### Carbofuran Induced the Phospholipid in Liver

**Figure 1:**
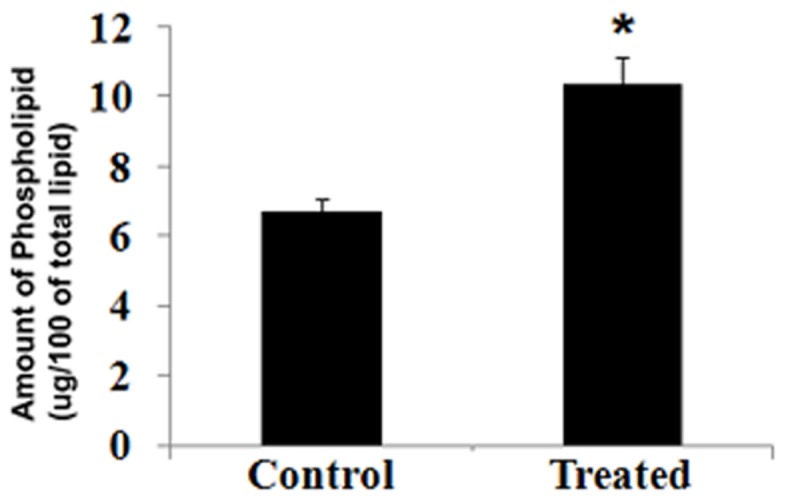
Treatment with carbofuran increased phospholipid in catfish livers. Injection of carbofuran and extraction of liver from catfish were performed according to ‘Materials and methods’. Total lipid was isolated from membrane fraction of liver, and phospholipid was estimated from total lipid. * indicates that the value is statistically significant, at *p*<0.05.

Upon treatment with carbofuran, the amount of total phospholipid increased significantly in the liver compared to vehicle-treated fish (Fig. 1). Phospholipid has been used to reconstitute the enzymatic activity of different purified P450 proteins, including P4501A and P4503A [32], [33], [34], but an intriguing question remains: How does increased phospholipid influence enzymatic activity of P4501A? Based upon the light of research of Agarwal et al., it can be conceivable that phospholipid may alter the structural conformation of purified protein which in turn augments the enzymatic activity.

### CF- induced Cytochrome P4501A Protein with EROD Activity was Purified from Catfish Liver by Using DE-52 and Hydroxyapaptite Column

**Figure 2:**
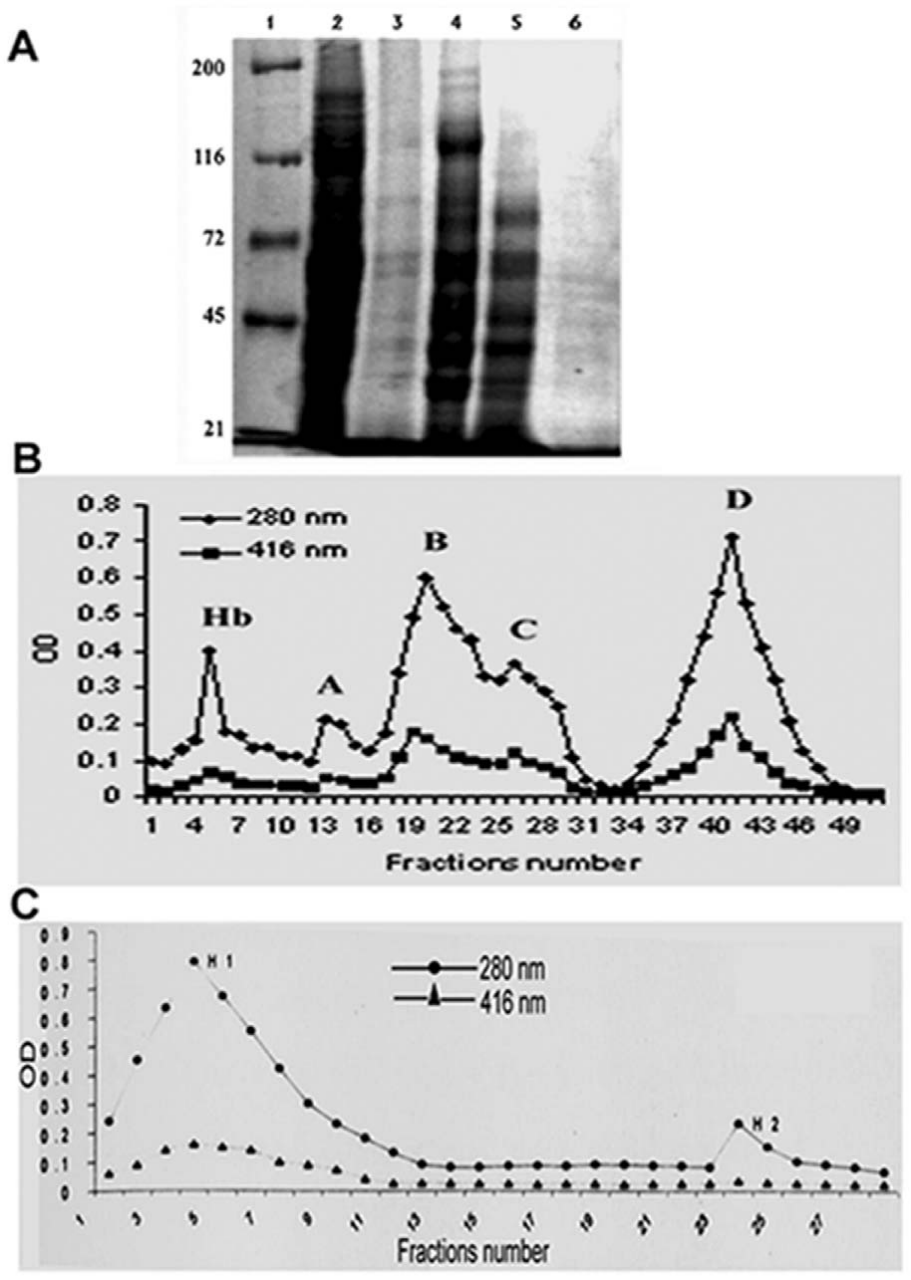
SDS-PAGE electrophoresis and chromatogram of eluted protein. A. SDS-PAGE (8%) was performed of polyethylene glycol (PEG) fractionated protein from solubilized microsome. Lane1, marker; lane 2, microsome; lane 3, solubilized microsome with CHAPS and sodium-cholate; lanes 4–6; solubilized microsomal protein with 10, 20, and 30% PEG, respectively. **B.** Chromatogram of eluted protein from DE-52 column. Protein was eluted from the column by linear gradient of KCl, and 10-ml fractions were collected. Four conspicuous peaks in terms of protein content measured at 280 nm were observed in the eluate as; Hb, A, B, C, and D (upper graph). Fractions were pooled, and heme content was tested at 416 nm (lower graph). **C.** The pooled fractions of D peak were added to the equilibrated hydroxyapatite column. The protein was eluted from the column by step gradient of phosphate buffer, and 5-ml fractions were collected. Fractions were pooled. There were two peaks, H1 and H2, in terms of protein content (upper graph). The lower graph represents the heme content of the pooled fractions.

Using CF-treated fish livers, we purified the cytochrome P4501A protein enriched in EROD activity. SDS-PAGE analysis of solubilized microsome after PEG-cut showed that 20% was the most suitable for availability of P450 protein (Fig. 2A). Therefore, protein obtained at 20% PEG was collected and loaded onto a DE-52 column. Four pooled fractions (Fraction-A, Fraction-B, Fraction-C, and Fraction-D) eluted from the DE-52 column were collected and used to measure specific activity (Fig. 2B). Fraction-D demonstrated the maximum ratio of cytochrome P450 protein when tested for heme and protein, respectively (Fig. 2B, lower graph)**.** In the reconstitution study, Fraction-D showed maximum reactivity to 7-ethoxyresorufin in comparison to any other fraction (Table 1).

**Table 1:** EROD activity of various fractions in reconstituted system.

Column	Sample	EROD Activity (pmol/min/mg protein)
DE-52	Microsome	606.76±12.45
	Fraction-A	ND
	Fraction-B	ND
	Fraction-C	104.55±6.89
	Fraction-D	283.83±11.67
Hydroxyapatite	Fraction-H1	202.23±11.23
	Fraction-H2	ND

EROD activity was reconstituted from fractions of various column eluates described in ‘Materials and methods’. Each value represents the mean ± SE from at least three observations. ND represents ‘Not Detected’.

Fractions-A and B contained minimum or no detectable activity, whereas Fraction-C demonstrated modest activity compared to Fractions-A and B (Table 1). Fraction-D which demonstrated the highest specific content and catalytic activity in the reconstituted system, was recovered and used in the hydroxyapatite column for further purification. In the hydroxyapatite column (Fig. 2C), two conspicuous peaks were obtained in terms of protein and heme absorbance at 280 nm and 416 nm wavelength; however, the second peak (H2) had a negligible protein amount (Fig. 2C, lower graph). The first peak from the hydroxyapatite column (H1) showed maximum activity to 7-ethoxyresorufin in the reconstitute system, and as expected, no such activity was detected for the H2 fraction (Table 1).

### Capillary Electrophoresis Showed the Highest Purity of the Cytochrome P4501A Protein

**Figure 3:**
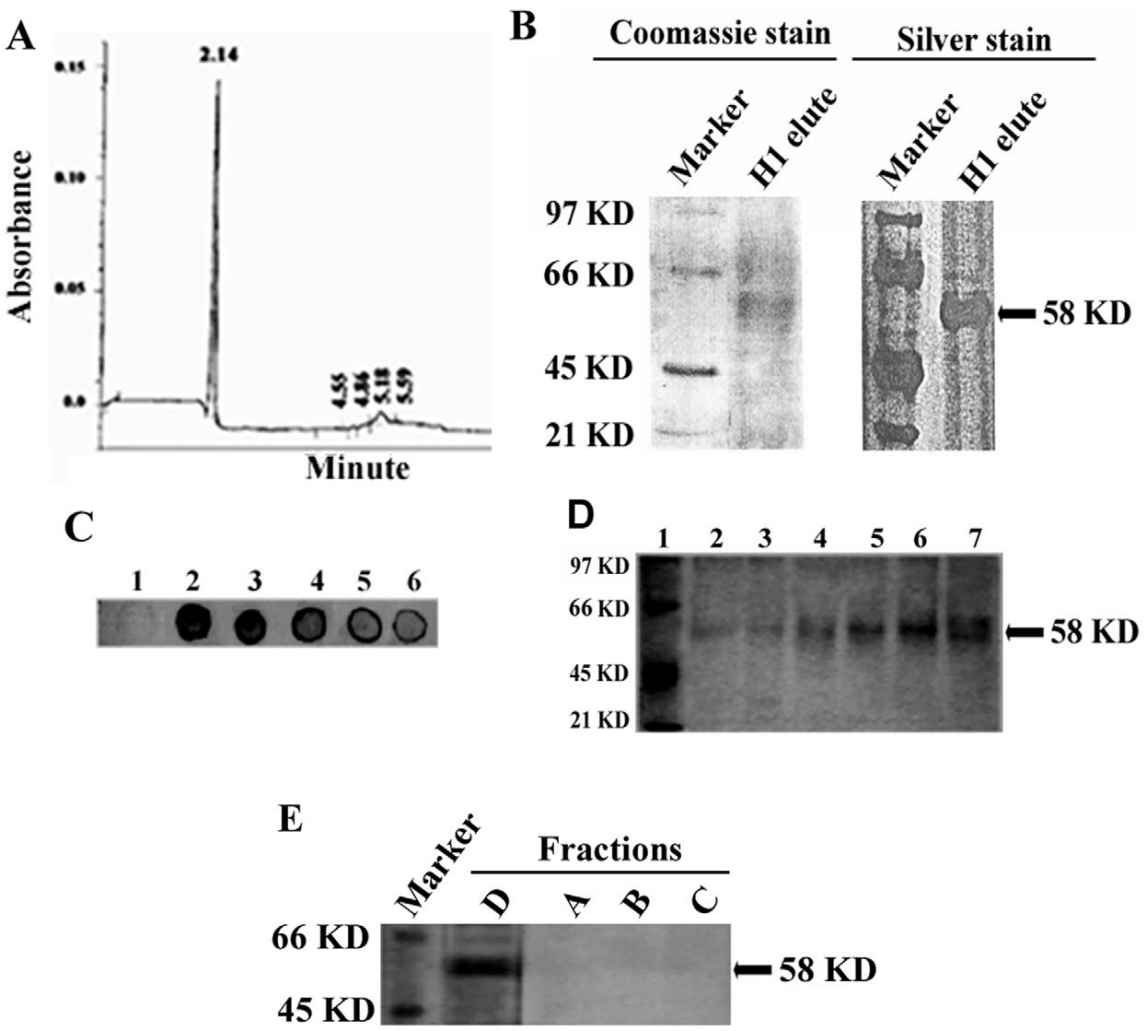
Biochemical characterization of purified protein. A. Capillary electrophoresis of purified fraction D was performed using amine capillary column 57 cm long as described in ‘Materials and methods’. The amount of protein added to the column was 1 µg. The result showed only a single peak at 2.14 min, indicating the presence of a protein of a single molecular weight. **B.** Coomassie and silver stain of H1 eluate was performed upon resolving the protein in 8% SDS-PAGE according to the procedure mentioned in ‘Materials and methods’. **C**. Dot-blot of purified fraction D using the polyclonal antibody against rabbit**.** 1, 2, 3, 4, 5, and 6 indicated BSA, 20, 10, 5, 2, and 1 µg of purified protein, respectively. **D**. Induction of cytochrome P4501A1 in carbofuran-treated hepatocytes. Cultured catfish hepatocytes were treated with different doses of carbofuran for 24 h. Lysate was made, and protein was run on 8% SDS-PAGE gel. Immunoblot was performed as described in ‘Materials and methods’ using the antibody against purified protein. Lane1, marker; lane 2, control; lanes 3–7, microsomal protein obtained from hepatocytes treated with various doses of CF: 0.1, 1.0, 10, 100, and 1000 nM, respectively. **E.** Immunoblot was performed by running all the fractions in 8% SDS-PAGE. The antibody raised against fraction D was used as a primary antibody. The rest of the procedure was followed as described in ‘Materials and methods’.

The purity of the H1 eluate was further checked by capillary electrophoresis with a loading amount of 1 µg. The profile showed a major and sharp peak at 2.16 min with optical density of 0.15 at 280 nm. No secondary, or so-called noise, peak was observed, meaning that the protein added in that column was a single molecule with a homogeneous mass (Fig. 3A). In the SDS-PAGE analysis, the H1 eluate revealed pure protein with a presumptive molecular mass of 58 KD determined by Gel-Doc software (Bio-Rad, Tokyo, Japan) (Fig. 3B). Data of both coomassie and silver staining supported the observation of capillary electrophoresis demonstrating a major single peptide at around 58 KD. Upon confirming the molecular identity of the H1 eluate, we decided to develop an antibody against the purified protein by using rabbit (Fig. 3B).

### Dot-blot and Western Blot Confirmed the Presence of Specific IgG in the Serum against the Purified Protein

Dot-blot analysis showed a gradual increase in density, a result that agreed with linear concentration of the purified protein adsorbed on the nitrocellulose membrane. No cross-reactivity was detected for the antibody with BSA (Fig. 3C). ELISA data also supported the reactivity of the antibody to purified protein (data not shown). Therefore, western blot was performed using the rabbit serum to test the induction of cytochrome P4501A protein containing EROD activity from the lysate of cultured hepatocytes that was treated with different doses of CF. The densitometry of western blot indicated a linear relationship of the bands with the CF doses (Fig. 3D).

### Identity of Fractions A, B, and C

We examined whether there are structural similarities in any motifs of fractions A, B, C with fraction D by immunoblot. All of those fractions were run into 8% SDS-PAGE and immunoblotted using the antibody raised against D. Data demonstrated that none of the fractions except D reacted with the antibody (Fig. 3E).

### Enzymatic activity of Cytochrome P4501A in the Reconstitution System Correlated as a Function of DLPC Concentration and Presence of α-helicity

**Figure 4:**
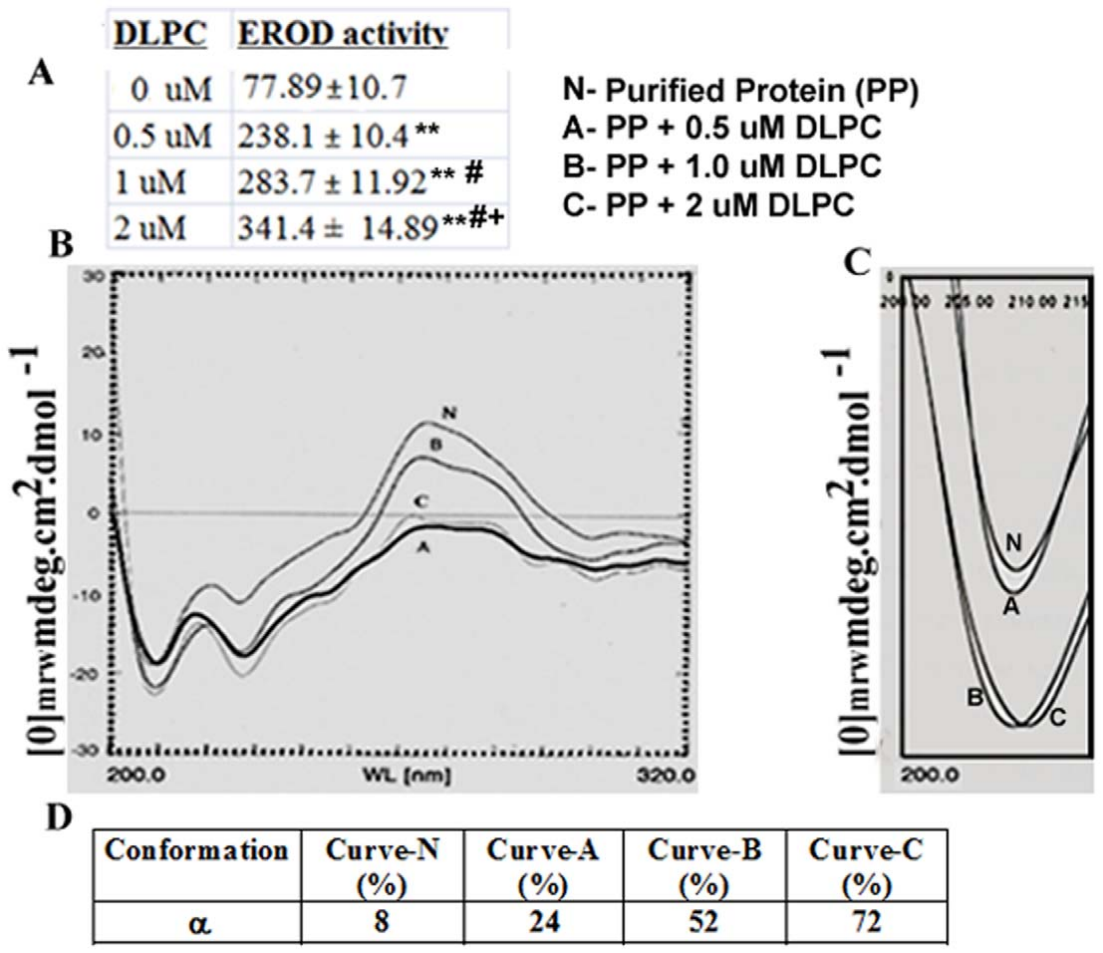
Reconstitution of purified protein with DLPC and circular dichroism (CD). A. Purified protein was sonicated with three different concentrations of DLPC (0.5, 1.0, and 2 µM), and reconstitution was done as mentioned in ‘Materials and Methods’. EROD activity was measured from reconstituted protein. ** values are significant at *p*<0.01 compared to 0 uM of DLPC. # values are significant at *p*<0.05 compared to 0.5 uM of DLPC.+values are significant at *p*<0.05 compared to 1.0 uM of DLPC **B.** Circular dichroism (CD) from reconstituted protein was performed. α helicity was determined from curves by the method of Greenfield and Fasman (1969). Representative curves from three observations are presented. N indicates purified protein (1 ug), and A, B, and C indicate those curves sonicated with 0.5, 1.0, and 2.0 uM of DLPC with the same amount of purified protein. **C.** An amplified view of a representative curve (not the 4 **B**) at 200–225 nm portions as the hump at 208 nm indicates the α-helicity. **D.** α-helicity was calculated from curves in different conditions. N, A, B, and C indicate the purified protein, and DLPC inserted purified protein at concentrations of 0.5, 1.0, and 2.0 uM, respectively.

The result of CD suggested that purified protein enriched with random coil in its conformation had been regaining α-helicity at a function of DLPC concentration; however, a synchronous loss of random coil clearly appeared in its conformation (Fig. 4B, 4D). The α-helicity of curves A, B, and C increased significantly compared to the curve of the only purified protein (curve N) (Table 2). The amplitude of α-helicity in the presence of DLPC was highly correlated with the enzymatic activity of the purified protein in the reconstitution system. Insertion of 1 uM and 2 uM of DLPC showed significantly higher (*p*<0.05 and *p*<0.01, respectively) EROD activity compared to 0.5 uM DLPC (Fig. 4A).

**Table 2:** Analysis of variance (ANOVA) of CD data using the statistical software SAS (Cary, NC).

α-Conformations	
Comparison	p-value
N Vs A	0.011*
N Vs B	0.0022**
N Vs C	0.00054**
A Vs B	0.014*
A Vs C	0.0020**
B Vs C	NS

Each attempt was replicated at least three times, and values of three observations for each point were considered for statistical analysis.

* indicated the values are significant at p<0.05;

** indicated the values are significant at p<0.01 level; NS, not significant.

## Discussion

Polycyclic aromatic hydrocarbons (PAH) pose a serious threat to the environment for their multipotent toxicities on the biosphere [35]. These compounds are reportedly implicated in altered regulation of CYP1A expression that is responsible for the detoxification of lipophilic hydrocarbons in the liver of aquatic vertebrates [1], [36], [37]. Based on its chemical structure that contains the aromatic benzofuran nucleus with aliphatic methyl groups as side chains, carbofuran can be categorized as a polycyclic aromatic hydrocarbon [23]. In a previous study, we observed that P4501A was induced by de novo synthesis to combat the presence of aromatic hydrocarbon compounds, such as, carbofuran or β-napthoflavone in the hepatocytes of catfish livers as evidenced by an increase in enzymatic activity of EROD [22]. Carbofuran induced the phospholipid in the membrane of hepatocytes which might be involved in activating a signaling cascade, including protein kinase C (PKC) or heat shock protein 70 (HSP70) [38]. The role of phospholipid in the activation of enzymatic activity of membrane-bound protein, including cytochrome P450 has been studied by many investigators [7], [8], [10], [39], [40], [41]. Many phospholipids, such as, dilaurylphosphatidylcholine (DLPC) and dilaurylphosphatidylserine (DLPS) have been used to reconstitute the enzymatic activity of cytochrome P450 protein [3], [33], [41], [42]. The underlying molecular mechanism of regaining the enzymatic activity of purified cytochrome P450 in the presence of phospholipid is partially understood. All these reports indicated the ability of phospholipid to influence structural conformation involving the enzyme [43], [44], [45]. Before looking into the structural data of purified protein, let us discuss different critical steps of purifying carbofuran-induced protein enriched with EROD activity from fish livers. Polyetheleneglycol (PEG) fractionation helped enriching the specific content of cytochrome P450 more than 2-fold, which facilitated in exclusion of many unwanted proteins from the population as shown in SDS-PAGE (Fig. 2). In our study, the hydroxyapatite column eluate appeared as a single molecular mass that was evidenced by capillary electrophoresis and SDS-PAGE data (Fig. 3). This fraction was reactive to 7-ethoxyresorufin and demonstrated the highest catalytic activity compared to the other fractions in the reconstituted system (Table 1). The antiserum against that protein in rabbit demonstrated cross-reactivity with Fraction-D from the DE-52 column. SDS-PAGE and immunoblot indicated the tentative molecular weight of carbofuran-induced cytochrome P450 protein to be ∼58 KD. Several other workers also demonstrated a protein band around 58 KD from different fish treated with inducers like β-napthoflavone and fish collected from naturally hydrocarbon-polluted water [5], [46]. The results suggested that Fraction-D from the DE-52 column was the main cytochrome P450 protein induced by carbofuran in catfish livers. This P450 might be involved in metabolization of this pesticide. The dose dependency of EROD activity from carbofuran -treated hepatocytes was clearly reflected in western blot analysis (Fig. 3D). We investigated the identity of these 3 fractions A, B, and C by western blot using the antibody raised against Fraction-D. Data demonstrated that the antibody of Fraction-D failed to bind other fractions, such as, fraction A, B, and C, which indicated the distinct structural identity of each isozymes. This may be the reason of why these fractions eluted out in different salt concentration from the DE-52 column (Fig. 3E).

Based upon CD data, it appeared that the structural conformation of purified Fraction-D varied significantly depending on the level of surrounding lipid environment. As cytochrome P450 is a membrane-bound protein, and its active conformation or catalytic activity depends on the lipid environment. The lipid moiety of the membrane always has major influences on the tertiary and quaternary conformations of the membrane-bound protein for its catalytic activity [10], [47]. Our CD data has shed light on the mechanism of how the purified protein restores its catalytic activity in the lipid microenvironment by altering the structural conformation in the reconstituted system. The change in structural conformations, such as, the α-helix, was found to be related to catalytic activity and amount of lipid in the reaction mixture (Table 2). It was reported that total lipid, including phospholipid in liver was elevated when an animal was exposed to carbofuran in laboratory conditions and natural environmental condition [23], [24], [48]. Our circular dichroism data have elucidated the mechanism of how this elevated lipid spectrum influenced the catalytic activity of P450 enzyme in vivo. The role of lipid-lipid bilayer in formation of proper membrane topology of P450 protein and its catalytic activity have already been documented [49], [50]. In our investigation, adding graded lipid to the buffer helped in gradual regaining of the α-helix which might one of the cause of elevated catalytic activity of this protein (Fig. 4A). A proper localization and topology are also required for NADPH-P450 reductase that acts as an electron donor to the P450s protein [7], [8], [51]. In our model, an appropriate structure contributed by more lipid in the reaction mixture could be involved with the facilitated interaction between the P450 and NADPH reductase, which may responsible for enhanced enzymatic activity of purified protein.

This study also elucidates the underlying mechanism of how enriched phospholipid microenvironment contributes in accelerated degradation of incoming substrate, such as, pesticides, drugs, and internal hydrophobic metabolites. Recent reports indicate that multivitamins and phospholipids protect the hepatocytes from steroid induced toxicity; however, abnormal lipogenesis in liver for obese people may disrupts the homeostasis of endoplasmic reticulum resulting an attenuated detoxification rate [52], [53]. Many reports unequivocally indicate that dietary lipid invariably influences the detoxification potential of metabolizing enzymes [54], [55], [56], [57]. Thus, our study clearly correlates the significance of enhanced phosphorlipid in the liver in physiological conditions which may be associated with orchestrating cytochrome P450’s enzymatic activity by influencing the structural and functional scaffold during detoxification.
